# Using state claims data to explore first-line antibiotic prescribing for acute respiratory conditions—Minnesota, 2018–2019

**DOI:** 10.1017/ash.2023.277

**Published:** 2023-09-29

**Authors:** Mari Freitas, Ashley Fell, Susan Gerbensky Klammer, Ruth Lynfield, Amanda Beaudoin

## Abstract

**Background:** Nationally, >30% of all outpatient antibiotics are unnecessary or inappropriate, and only 52% of outpatients with sinusitis, otitis media, or pharyngitis receive recommended first-line antibiotics. The Minnesota All Payer Claims Database (MN APCD) collects medical claims, pharmacy claims, and eligibility files from private and public healthcare payers. We analyzed claims to describe overall and firstline antibiotic prescribing for acute bronchitis, adult acute sinusitis, and pediatric patients. **Results:** We analyzed 3,502,013 respiratory events from 1,612,501 members. Acute bronchitis accounted for 179,723 events (5.1%), acute sinusitis accounted for 236,901 adult events (10%), and otitis media accounted for 232,226 pediatric events (19%). Also, 73,385 bronchitis diagnoses (~40%) had no associated antibiotic. Antibiotics were associated with 199,445 adult sinusitis events (84.2%), of which 89,386 (44.8%) were firstline antibiotics, and 190,962 pediatric otitis media events (82.2%), of which 126,859 (66.4%) were firstline antibiotics. Common antibiotic classes used when a firstline drug was not selected were macrolides (28.9%) and tetracyclines (26.8%) for adult acute sinusitis and cephalosporins (61.4%) and macrolides (30.6%) for pediatric otitis media. Compared to the least vulnerable quartile, the most vulnerable social vulnerability index (SVI) quartile had lower odds of receiving firstline antibiotics for adult acute sinusitis if antibiotics were prescribed (OR, 0.90; 95% CI, 0.87–0.94) and higher odds of receiving firstline antibiotics for pediatric otitis media if antibiotics were prescribed (OR, 1.16; 95% CI, 1.12–1.21). **Conclusions:** Improvement is needed in avoiding antibiotics for acute bronchitis and selecting firstline drugs for sinusitis and otitis media. Additional analyses adjusting for demographic, geographic, and prescriber factors are planned to better understand differences in prescribing appropriateness among Minnesotans.

**Disclosures:** None

